# Determination of diagnostic and predictive parameters for vertical mandibular invasion in patients with lower gingival squamous cell carcinoma: A retrospective study

**DOI:** 10.1097/MD.0000000000032206

**Published:** 2022-12-09

**Authors:** Takahiro Shimizu, Mai Kim, Citra R.A.P. Palangka, Mai Seki-Soda, Masaru Ogawa, Yu Takayama, Satoshi Yokoo

**Affiliations:** a Department of Oral and Maxillofacial Surgery, and Plastic Surgery, Gunma University Graduate School of Medicine, Maebashi, Gunma, Japan; b Department of Diagnostic Radiology and Nuclear Medicine, Gunma University Graduate School of Medicine, Maebashi, Gunma, Japan; c Department of Diagnostic Pathology, Gunma University Graduate School of Medicine, Maebashi, Gunma, Japan.

**Keywords:** ^18^F-FDG PET, lower gingival squamous cell carcinoma, surgical method, vertical mandibular invasion, volume-based parameter

## Abstract

Vertical mandibular invasion of lower gingival squamous cell carcinoma (LGSCC) determines the method of resection, which significantly affects the patient’s quality of life. Therefore, in mandibular invasion by LGSCC, it is extremely important to monitor progression, specifically whether invasion is limited to the cortical bone or has progressed to the bone marrow. This retrospective study aimed to identify the diagnostic and predictive parameters for mandibular invasion, particularly vertical invasion, to enable appropriate selection of the method of mandibular resection. Of the patients who underwent surgery for LGSCC between 2009 and 2017, 64 were eligible for participation in the study based on tissue microarrays (TMA) from surgical specimens. This study analyzed morphological features using computed tomography (CT), and metabolic characteristics using maximum standardized uptake value (SUVmax), peak value of SUV (SUVpeak), metabolic tumor volume (MTV), and total lesion glycolysis (TLG). Moreover, immunohistochemical analysis of proteins, including parathyroid hormone-related protein (PTHrP), interleukin-6 (IL-6), E-cadherin, and programmed cell death-1 ligand 1 (PD-L1), was performed. Statistical analysis was performed using univariate logistic regression analysis with the forward selection method. The present study showed that MTV (≥2.9 cm^3^) was an independent diagnostic and predictive factor for positivity of mandibular invasion. Additionally, TLG (≥53.9 bw/cm^3^) was an independent diagnostic and predictive factor for progression to bone marrow invasion. This study demonstrated that in addition to morphological imaging by CT, the volume-based parameters of MTV and TLG on fluorine-18 fluorodeoxyglucose positron emission tomography were important for predicting pathological mandibular invasion in patients with LGSCC. A more accurate preoperative diagnosis of vertical mandibular invasion would enable the selection of appropriate surgical procedure for mandibular resection.

## 1. Introduction

**Figure 1.**
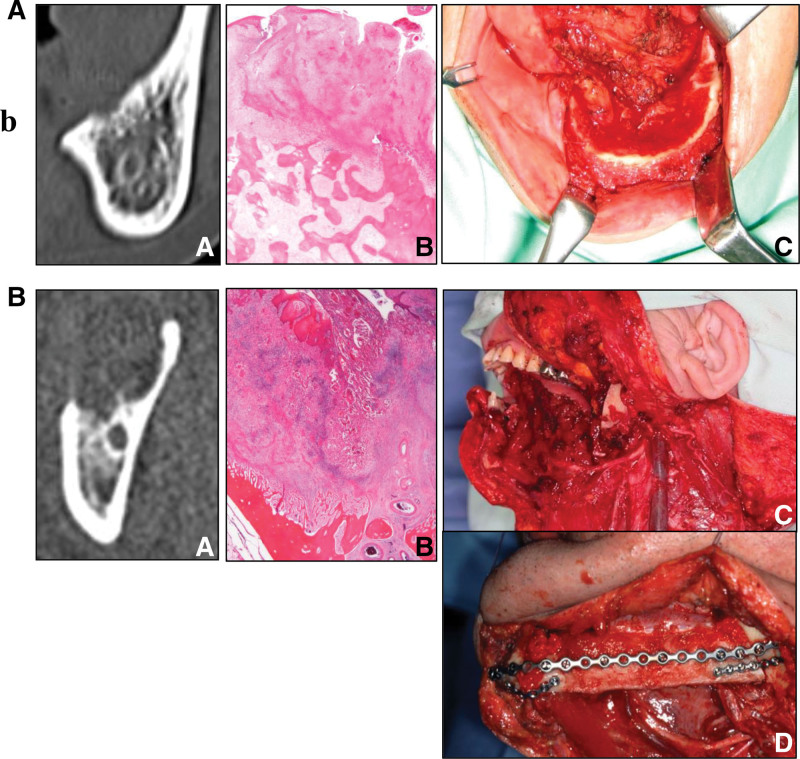
**Vertical mandibular invasion and operation methods of lower gingival squamous cell carcinoma.** If no mandibular invasion is diagnosed, bone scraping or corticotomy is performed to achieve safe margins. Marginal mandibular resection is required for cortical bone invasion alone, and segmental mandibular resection if bone marrow invasion is diagnosed. Patients undergoing segmental mandibular resection requires reconstructive surgery. (A) Invasion limited to the cortical bone: a) CT imaging, b) pathological image, and c) marginal mandibular resection. (B) Invasion of the bone marrow: a) CT imaging, b) pathological image, c) segmental mandibular resection, and d) oromandibular reconstruction with vascularized free fibular flap. CT = computed tomography.

Since the lower gingiva and mandible are in direct contact via the periosteum, lower gingival squamous cell carcinoma (LGSCC) can easily invade the mandible. Determining the appropriate extent of mandibular resection, which affects treatment outcomes such as local control and prognosis of LGSCC, is crucial.^[1]^ If no mandibular invasion is diagnosed, bone scraping or corticotomy can be performed to achieve safe margins. Moreover, marginal mandibular resection is indicated in cortical bone invasion only, and segmental mandibular resection if bone marrow invasion is diagnosed. Patients undergoing segmental mandibular resection requires reconstructive surgery, which is not only a highly invasive surgery, but also significantly influences the patient’s post-operative quality of life (QOL). Therefore, in mandibular invasion by LGSCC, it is extremely important to determine the progression, whether bone marrow invasion is present or if invasion is limited to cortical bone (Fig. 1).

Mandibular invasion in patients with LGSCC has been diagnosed using a combination of morphological imaging modalities such as panoramic radiography, computed tomography (CT), and magnetic resonance imaging (MRI).^[2,3]^ We have previously shown that fluorine- 18 fluorodeoxyglucose positron emission tomography (^18^F-FDG PET), a metabolic imaging technique for assessing tumor activity, is more sensitive than MRI for evaluating bone marrow invasion in patients with gingival cancer, although MRI is superior to in specificity.^[4]^

^18^F-FDG PET is a useful modality for assessing prognosis and treatment efficacy as well as for predicting recurrence, as it reflects metabolic activity related to tumor viability and proliferation. The standardized uptake value (SUV) is widely used as a semi-quantitative parameter of ^18^F-FDG accumulation in lesions. In recent years, the peak value of SUV (SUVpeak) has replaced the maximum value of SUV (SUVmax) in terms of reproducibility and stability. Additionally, analyses of the metabolic tumor volume (MTV) and total lesion glycolysis (TLG) by ^18^F-FDG PET have been used to investigate the prognostic parameters in patient with various solid malignancies including oral squamous cell carcinoma (OSCC).^[5–8]^ Although the usefulness of metabolic parameters has been reported for the diagnosis of mandibular invasion in OSCC, all previous studies have used SUVmax alone,^[9,10]^ and no studies have applied volume-based metabolic parameters such as MTV and TLG. Furthermore, these metabolic imaging diagnoses are mainly focused on invasion of the bone marrow with obvious cortical bone destruction and horizontal intramedullary extension. Therefore, previous studies have investigated the association between bone marrow invasion and prognosis but not the selection of surgical methods.

The relationship between osteoclast-inducing cytokines released by carcinomas, such as parathyroid hormone-related protein (PTHrP), tumor necrosis factor-α (TNF-α), interleukin (IL)-6, and IL-11, and bone invasion in patients with OSCC has been reported.^[11–14]^ However, there have been no reports on the relationship between vertical carcinoma invasion, precisely, progression to the bone marrow, or limitation to the cortical bone.

Programmed cell death-1 ligand 1 (PD-L1), which is expressed as a cell surface molecule on various cancer cells, binds to programmed cell death-1 (PD-1) expressed on activated T-cells as an immune checkpoint molecule and inhibits their activation. Therefore, PD-L1 expression, an important indicator of tumor immune resistance, has been reported to be a prognostic predictor of various carcinomas, including OSCC.^[15–19]^ A relationship between invasive ability and PD-L1 expression in OSCC has been reported previously.^[19]^ However, the relationship between bone invasion and PD-L1 expression in patients with OSCC, particularly LGSCC, remains unclear.

The purpose of this retrospective study was to determine the relationship between pathological vertical invasion, precisely, progression to bone marrow invasion or limitation to the cortical bone in patients with LGSCC and clinical factors, morphological and metabolic imaging factors, and expression of osteoclast-induced cytokines and PD-L1 at the tumor-bone interface. Further, we examined the applicability of these results in selecting the appropriate method of vertical mandibular resection, from among bone scraping/corticotomy, marginal resection, and segmental resection.

## 2. Patients and Methods

### 2.1. Patients

Among the 80 patients diagnosed with LGSCC and underwent surgery at the Department of Oral and Maxillofacial Surgery, Gunma University Hospital from July 2009 to March 2017, 64 were included in the study. This study conformed to the Declaration of Helsinki and was approved by the Institutional Research Committee at Gunma University (IRB number: HS2018-108). Consent was obtained from the enrolled patients using the opt-out method.

### 2.2. Analysis of morphological (CT imaging) and pathological bone invasion

First, we investigated the concordance between morphological and pathological diagnoses of mandibular invasion on CT imaging. Subsequently, we assessed the concordance between vertical invasion (progression to the bone marrow or limitation to the cortical bone) on CT imaging and pathological invasion. Based on these analyses, the usefulness and limitations of CT imaging in selecting the surgical method were determined. This involved calculating the prevalence, sensitivity, specificity, positive predictive value (PPV), negative predictive value (NPV), false cortical bone invasive rate (under-surgery rate), and false bone marrow invasive rate (over-surgery rate) of CT imaging. CT was performed using a SOMATOM Definition Flash (Siemens Healthcare Co. Ltd., Forchheim, Germany) and a Light Speed VCT (GE Healthcare Co. Ltd., Tokyo, Japan). CT imaging diagnosis was assessed by 2 radiologists and 3 oral and maxillofacial surgeons, and pathological invasion was assessed by 2 pathologists and 2 oral and maxillofacial surgeons using postoperative hematoxylin and eosin (H-E) stained specimens in a blinded manner.

### 2.3. Metabolic imaging analysis

For PET, all patients fasted for 6 hours prior to imaging. After intravenous administration of FDG (4.0 MBq/kg), patients were allowed to rest for 60 minutes before imaging. The images were analyzed by a certified PET nuclear medicine physician with clinical experience in head and neck imaging using the analysis software workstation syngo via (Multimodality Workplace, Siemens Healthcare GmbH, Erlangen, Germany). SUVmax (Bq/mL) and SUVpeak (Bq/mL) were calculated as the imaging parameters of the SUV (Bq/mL). SUVmax is the maximum value of 1 pixel in a region of interest (ROI). Abnormal accumulation in LGSCC was manually set as the 3-dimensional volume of interest (VOI). The mean value of the SUV at a 1 cm^3^ voxel was measured in the VOI and the mean value, SUVpeak, was calculated. In addition, MTV and TLG were analyzed as 2 imaging parameters for FDG accumulation in the whole tumor. The metabolic tumor volume above the SUV value of 2.5 Bq/kg threshold was measured as MTV (cm^3^).^[20,21]^ TLG was calculated as the MTV multiplied by the mean value of the SUV (SUVmean), which reflects both glucose metabolic activity and tumor volume.^[4–8]^

### 2.4. Analysis of mode of invasion of carcinoma

Pathological factors used to determine the appropriate surgical method were investigated using the infiltrating factor (INF), which is used to evaluate gastrointestinal cancers, such as those of the esophagus, stomach, and colon. With respect to tumor INFs in the surrounding tissue, INFa was defined as a tumor that displayed expanding growth with a distinct border from the surrounding tissue, INFb as a tumor that showed an intermediate pattern between INFa and INFc, and INFc as a tumor that displayed infiltrative growth with no distinct border from the surrounding tissue.^[22]^ In addition, tumor budding, a pathological finding in which cancer cells invaded the stroma either as single cells or diffusely in small nests comprising <5 cells at × 200 magnification, was also important.^[23–25]^ Regarding the biopsy findings, even if the primary lesion was pathologically INFb, it was treated as INFc if it was poorly differentiated or had a tumor budding score of 5 or more. Slides were evaluated by 2 pathologists and 2 oral and maxillofacial surgeons in a blinded manner.

### 2.5. TMA and immunohistochemistry

To limit evaluation to the deepest part of the carcinoma, a non-demineralized specimen of the deepest part of the tumor found in the mandible was extracted with a 2 mm diameter punch tip. The prepared tissue microassay (TMA) was sectioned at a thickness of 2.5 *μ*m. Thereafter, the sections were prepared with H-E staining.^[26,27]^

In this study, immunohistochemistry (IHC) of proteins (Table 1) in the TME was performed using a 2-step staining method. The peroxidase-anti-peroxidase complex method was used for immunostaining with PTHrP, IL-6, E-cadherin, and PDL-1 antibodies. The sections were treated with 3% hydrogen peroxide in methanol at room temperature for 15 minutes to inhibit the endogenous peroxidase, and were then incubated with IL-6, PTHrP, E-cadherin and PDL-1 antibodies at 1:2000, 1:1000, 1: 100, and 1:1000 dilution overnight at 4℃ respectively. After washing, the sections were incubated with secondary antibodies for 30 minutes at room temperature, and the slides were sealed.

**Table 1:** Immunohistochemical staining.

Primary antibodies	Source	Dilution	Clone	Purpose
PTHrP	Yanaihara Institute Inc.	1:1000	Rabbit polyclonal antibody	• Cytokine released by cancer cells. • PTHrP induces osteoblasts to express the RANKL. Binding of RANKL to the RANK receptor of osteoclast precursor cells induces NFATc1, a meister transcription factor that integrates RANKL-activated signals, promoting osteoclast differentiation.
IL-6	Rockland Inc.	1:2000	Rabbit polyclonal antibody	• Inflammatory cytokines released by cancer cells. • Since stimulation by IL-6 and TNF-α induces bone-resorbing multinucleated giant cells, it is possible that some mechanisms of the bone-resorptive effects of IL-6 are not mediated by the RANK/RANKL pathway.
E-cadherin	Leica Biosystems Newcastle Ltd.	1:100	Mouse monoclonal antibody	• Epithelial cell adhesion molecules • One of the biomarkers associated with EMT.
PD-L1	Gene Tex Inc.	1:1000	Rabbit polyclonal antibody	• Biomarkers related to tumor immunity • By binding to PD-1, it suppresses the immune activity of T cells and promotes the growth of cancer cells. • In squamous cell carcinomas, there may be an association between PD-L1 expression and tumor activity and prognosis.

EMT = epithelial-mesenchymal transition, IL-6 = interleukin 6, NAFATc1 = nuclear factor-activated T cells c1, PD-1 = programmed death-1, PD-L1 = programmed cell death 1-ligand 1, PTHrP = parathyroid hormone-related protein, RANK = receptor activator of nuclear factor кB, RANKL = receptor activator of nuclear factor кB ligand, TNF-α = tumor necrosis factor-α.

**Figure 2.**
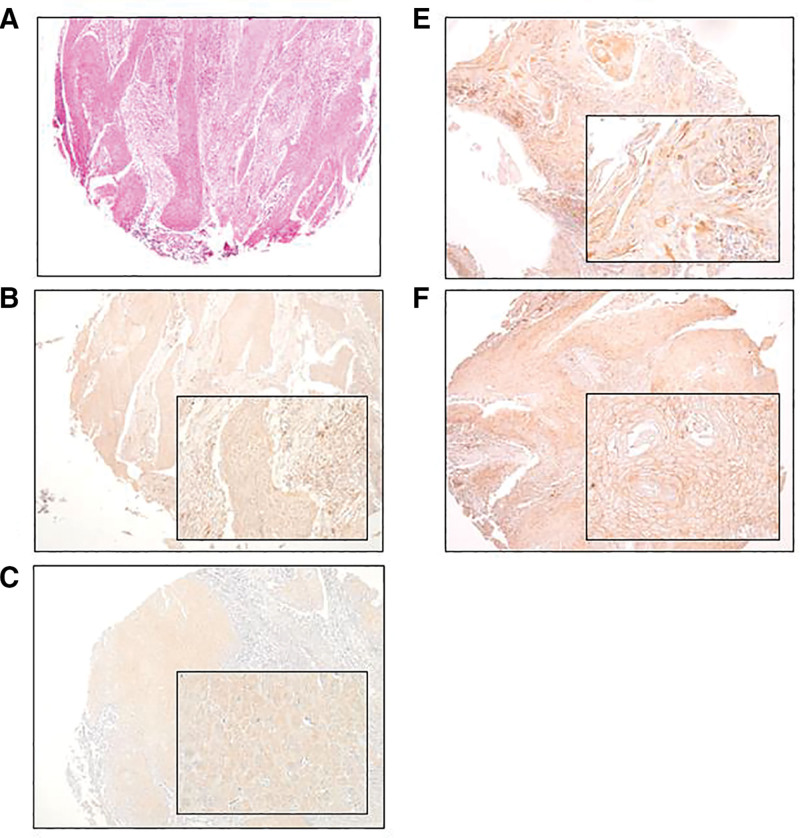
**Immunohistochemical analysis in tissue microarray.** (A) Haematoxylin and eosin staining (×40), (B) parathyroid hormone-related protein positive cytoplasm (×40 and ×200), (C) interleukin-6 positive cytoplasm (×40 and ×200), (D) E-cadherin positive cell membrane (×40 and ×200), and (E) programmed cell death-1 ligand 1 positive cell membrane (×40 and ×200).

Analysis of each staining in IHC was done according to the visual intensity of carcinoma cells. Slides were evaluated by 2 pathologists and 2 oral and maxillofacial surgeons in a blinded manner. IL-6 and PTHrP scores were calculated by considering tumor cells with staining of the cytoplasm in a 100 × field of view as positive. Regarding IL-6, scores 0 and 1 were classified as low expression and scores 2 and 3 as high expression (score 0: positive cell = 0%, score 1: positive cell < 25%, score 2: positive cell < 50%, score 3: positive cell: ≥ 50%).^[28]^ As for PTHrP scores, score 0 was defined as a percentage of positive cells less than 10%, while score 1 was ≥ 10%.^[26,27]^ E-cadherin and PD-L1 scores were calculated by considering tumor cells with staining of the cell membrane in the 100 × field of view. In terms of E-cadherin, scores 0 and 1 were classified as negative and scores 2 and 3 as positive; (score 0, positive cells = 0%; score 1, positive cells < 10%; score 2: positive cells ≤ 10%, and score 3, positive cells ≥ 50%).^[19]^ As for PD-L1, scores 0–2 were classified as low and scores 3–6 as high (score 1, positive cells < 1%; score 2, 1% ≤ positive cells < 5%; score 3, 5% ≤ positive cells < 10%; score 4, 10% ≤ positive cells < 25%; score 5, 25% ≤ positive cells < 50%) (Fig. 2).^[19]^

### 2.6. Statistical analysis

To assess the factors determining pathological mandibular invasion, the following parameters were analyzed: First, clinical factors including sex and tumor size (preoperative horizontal diameter). Second, pathological factors including tumor differentiation (WHO grade), mode of invasion (INF), osteoclast-induced protein expression (PTHrP, IL-6), cell adhesion molecule expression (E-cadherin), and immune checkpoint molecule expression (PD-L1) were also analyzed. Third, imaging factors comprising morphological (mode of bone resorption by CT) and metabolic factors (SUVmax, SUVpeak, MTV, and TLG) were analyzed.

**Figure 3.**
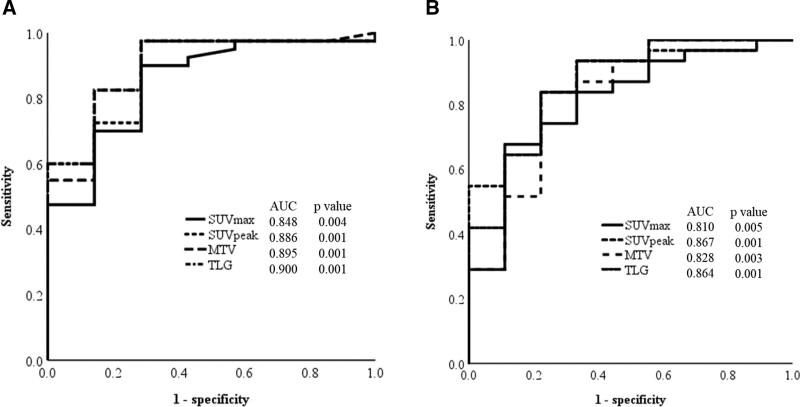
**Calculation of cutoff values for imaging parameters using receiver operating characteristic curves.** Calculation of metabolic parameter in pathological bone invasion positive or negative. (A). Calculation of metabolic parameter in vertical bone invasion (progression to the bone marrow or limitation to the cortical bone).

To compare the positive or negative pathological mandibular invasion, all parameters were subjected to univariate analysis using a cross table with the *χ*^2^ test for independence. Continuous values of imaging factors (SUVmax, SUVpeak, MTV, and TLG) were converted into binary values by calculating cutoff value using receiver operating characteristic (ROC) curve analysis (Fig. 3). Next, items with significant differences between the 2 groups (positive and negative pathological invasion) were calculated using logistic regression analysis with the forward-selection method. Patients with pathological mandibular invasion were further divided into 2 groups; progression to the bone marrow and limitation to the cortical bone, and differences between them were analyzed using the same method. A variance inflation factor (VIF) was calculated to eliminate multicollinearity between explanatory variables. Explanatory variables with VIF < 10, which indicates no multicollinearity, were used to endure that no variables had multicollinearity. Statistical analysis was performed with SPSS for Windows, ver.25 (IBM Corp., Armonk, NY). A *P* value < .05 was considered to denote significance.

## 3. Results

### 3.1. Patient backgrounds for determining the method of mandibular resection

The patient backgrounds are presented in Table 2. The analyzed factors influencing the vertical mandibular surgical approach for LGSCC were age, T-stage among the clinical factors, INF as a pathological factor, and mode of bone invasion assessed by CT imaging. The patients’ age ranged from 38 to 90 years, with a median age of 71 years. Twenty-five patients (39%) were staged T1 to T3, and 39 (61%) were T4a. Tumor sizes ranged from 8 to 65 mm, with a median size of 28 mm. Among the pathological factors identified through biopsy, INFc accounted for 40% of the total. The diagnosis of mandibular invasion was made by CT imaging, and of the 47 patients with mandibular invasion, invasion was limited to the cortical bone 8 (13%) patients, and had progresses to the bone marrow in 39 (61%) patients.

**Table 2:** Patient characteristics influencing the determination of the method of mandibular resection.

			Patients	
			(n = 64)	%
Clinical factors				
	Age/yrs (median)		38–90 (71)	
	Sex			
		Man	43	67
		Woman	21	33
	T-stage			
		1	7	11
		2	16	25
		3	2	3
		4a	39	61
	Tumour size/mm (median)		8–65 (28)	
Pathological factors	Tumour differentiation (WHO grade)			
		1	40	63
		2	20	32
		3	3	5
		4	0	0
	INF			
		a	10	15
		b	27	42
		c	26	40
Imaging factors	Bone invasion in CT			
		No invasion	17	26
		Limited to the cortical bone	8	13
		Progressed to the bone marrow	39	61

CT = computed tomography, INF = infiltration factor, WHO = world health organization.

### 3.2. Analysis of morphological bone invasion (CT imaging) and pathological bone invasion

Table 3 (A) shows the concordance between CT imaging and pathological diagnosis of mandibular invasion. Patients diagnosed with positive invasion on CT imaging were significantly pathologically diagnosed with positive invasion (*P* < .001). Prevalence, sensitivity, specificity, PPV and NPV of CT imaging for pathology were 79.7%, 84.3%, 69.2%, 91.5% and 52.9% respectively. A false negative rate (under-surgery rate) was observed in 15.7% of the patients and a false positive rate (over-surgery) in 8.5%.

**Table 3:** Analysis of morphological bone invasion (CT imaging) and pathological bone invasion.

**(A) Relationship between invasion and no invasion**								
	Pathological	Invasion (+)	Invasion (−)	Total		Odds ratio	95% CI	*P* value
CT imaging								
Invasion (+)		43	4	47	64	12.1	2.9–48.9	<.001*
Invasion (−)		8	9	17				
Prevalence (Positive pathological invasive rate) = 79.7&, PPV = 91.5%, Sensitivity = 84.3%, NPV = 52.9%, Specificity = 69.2%, False negative rate (under-surgery rate) = 15.7%, False positive rare (over-surgery rate) = 8.5%.								
**(B) Relationship between bone marrow invasion and cortical bone invasion (detailed analysis of 47 patients with invasion [+]**)								
	Pathological	Bone marrow	Cortical bone	Total		Odds ratio	95% CI	*P* value
CT imaging								
Bone marrow		33	6	39	47	9.2	1.7–48.9	
Cortical bone		3	5	8				
Prevalence (Bone marrow invasion rate) = 76.7%, PPV = 84.6%, Sensitivity = 91.7%, NPV = 62.5%, Specificity = 62.5%, False negative rate (under-surgery rate) = 8.3%, False positive rate (over-surgery rare) = 15.3%.								

CI = confidence interval, CT = computed tomography, NPV = negative predictive value, PPV = positive predictive value.

* *P* value < .05.

Table 3 (B) shows the concordance between the pathological diagnosis of vertical invasion (progression to the bone marrow or limitation to the cortical bone) in 47 patients diagnosed with mandibular invasion on CT imaging. Patients identified having progression to the bone marrow on CT imaging were significantly pathologically confirmed to have progression to the bone marrow (*P* = 0011). Prevalence (bone marrow invasive rate), sensitivity, specificity, PPV, and NPV of CT imaging for pathological bone marrow invasion were 76.7%, 91.7%, 62.5%, 84.9%, and 62.5% respectively. A false negative rate (under-surgery rate) was observed in 8.7% and a false positive rate (over-surgery) in 15.3% of the patients.

### 3.3. Diagnostic and predictive factors of the pathological bone invasion

#### 3.3.1. Diagnostic and predictive factors of pathological positive and negative bone invasion.

Table 4 shows the results of analysis of diagnostic and predictive factors of pathological mandibular invasion. Tumour size (*P* = .001), INF (*P* = .006), PTHrP (*P* = .026), SUVmax (*P* = .001), SUVpeak (*P* < .001), MTV (*P* < .001), and TLG (*P* < .001) were diagnostic and predictive factors in the univariate analysis. In the multivariate analysis of these factors (logistic regression analysis with the forward selection method), excluding SUVmax, which had a VIF > 10, MTV was an independent predictive factor (*P* = .001).

**Table 4:** Diagnostic and predictive parameters of pathological bone invasion.

		Univariable						Multivariable	
		Pathological bone invasion positive (n = 51)	Pathological bone invasion negative (n = 13)	Odds ratio	95% CI	*P* value	VIF	Odds ratio	*P* value
Clinical factors	Age (yrs)								
	≥72	21	9						
	<72	30	4	-	-	.07			
	Sex								
	Male	35	8						
	Female	16	5	-	-	.43			
	Tumor size (mm)								
	≥26.5	33	2						
	<26.5	18	11	10.1	2.0–50.5	.001*	1.2		
Pathological factors	Tumor differentiation (WHO grade)								
	3,4	3	0						
	1,2	47	13	-	-	.494			
	INF								
	c	25	1						
	a,b	25	12	12	1.4–99.3	.006*	1.2		
	PTHrP								
	+	44	9						
	−	1	3	14.7	1.3–157.5-	.026*	1.0		
	IL-6								
	High	25	3						
	Low	24	9	-	-	.105			
	E-cadherin								
	−	31	8						
	+	16	5	-	-	.505			
	PD-L1								
	High	34	8						
	Low	15	4	-	-	.555			
Imaging factors	SUVmax								
	≥6.1	36	2						
	<6.1	4	5	22.5	3.2–156.2	.001*	17.5		
	SUVpeak								
	≥3.8	39	2						
	<3.8	1	5	97.5	7.4–1279.8	<.001*	1.0		
	MTV								
	≥2.9	39	2					85.0	
	<2.9	1	5	97.5	7.4–1279.8	<.001*	1.5	(6.4–1118.9)	.001*
	TLG								
	≥10.1	39	2						
	<10.1	1	5	97.5	7.4–1279.8	<.001*	2.2		

CI = confidence interval, IL-6 = interleukin 6, INF = infiltration factor, MTV = metabolic tumor volume, PD-L1 = programmed death 1-ligand 1, PTHrP = parathyroid hormone-related protein, SUVmax = maximum value of SUV, SUVpeak = peak value of SUV, TLG = total lesion glycolysis, VIF = variance inflation factor, WHO = World Health Organization.

* *P* value < .05.

#### 3.3.2. Diagnostic and predictive factors of pathological vertical mandibular invasion (Progressive to the bone marrow or limitation to the cortical bone).

Table 5 shows the results of analysis of diagnostic and predictive factors of pathological vertical mandibular invasion (progressive to the bone marrow or limitation to the cortical bone) in 51 patients. Tumor size (*P* = .048), INF (*P* = .047), SUVmax (*P* = .004), SUVpeak (*P* = .001), MTV (*P* = .001) and TLG (*P = *.001) were diagnostic and predictive factors in the univariate analysis. In the multivariate analysis of these factors (logistic regression analysis with the forward selection method), excluding SUVmax, which had a VIF > 10, TLG was an independent diagnostic and predictive factor (*P* = .001).

**Table 5:** Diagnostic and predictive parameters of pathological vertical bone invasion (progression to the bone marrow or limitation to the cortical bone).

		Univariable					Multivariable	
		Bone marrow invasion (n = 39)	Cortical bone invasion (n = 12)	Odds ratio (95% CI)	*P* value	VIF	Odds ratio (95% CI)	*P* value
Clinical factors	Age (years)							
	≥80	6	4					
	<80	33	8	-	.169			
	Sex							
	Male	27	8					
	Female	12	4	-	.565			
	Tumor size (mm)							
	≥35.5	19	2	4.8				
	<35.5	20	10	(9.1–24.5)	.048*	1.1		
Pathological factors	Grade							
	3, 4	3	0					
	1, 2	35	12	-	.43			
	INF							
	c	22	3	4.1				
	a, b	16	9	(0.9–17.7)	.047*	1.1		
	PTHrP							
	+	34	20					
	−	0	1	-	.244			
	IL-6							
	High	17	8					
	Low	19	5		.376			
	E-cadherin							
	−	22	9					
	+	14	2	-	.185			
	PD-L1							
	High	26	8					
	Low	11	4	-	.54			
Imaging factors	SUVmax							
	≥12.0	21	1	16.8				
	<12.0	10	8	(1.8–153.3)	.004*	16.0		
	SUVpeak							
	≥7.1	26	2	18.2				
	<7.1	5	7	(2.8–114.5)	.001*	1.0		
	MTV							
	≥12.8	26	2	18.2				
	<12.8	5	7	(2.8–114.5)	.001*	1.4		
	TLG							
	≥53.9	26	2	18.2			22.7	
	<53.9	5	7	(2.8–114.5)	.001*	1.9	(3.4–150.8)	.001*

CI = confidence interval, VIF = variance inflation factor, WHO = World Health Organization, INF = infiltration factor, PTHrP = parathyroid hormone-related protein, IL-6 = interleukin 6, PD-L1 = programmed death 1-ligand 1, SUVmax = maximum value of SUV, SUVpeak = peak value of SUV, MTV = metabolic tumor volume, TLG = total lesion glycolysis.

* *P* value < .05.

#### 3.3.3. Decision analysis of MTV and TLG for diagnostic and predictive factors of bone invasion.

Table 6 (A) shows the results of decision analysis of the diagnostic and predictive parameters of pathological positive mandibular invasion for MTV ≥ 2.9 cm^3^ (cutoff values obtained by ROC analysis [Table 4, Fig. 3]). The results obtained were: sensitivity, 97.5%; specificity, 71.4%; PPV, 95.1%; and NPV, 83.3%. Table 6 (B) shows the results of the decision analysis of bone marrow invasion and cortical bone invasion for TLG ≥ 53.9 bw/cm^3^ (cutoff values obtained by ROC analysis [Table 5, Fig. 3]). The results obtained were: sensitivity, 83.9%; specificity, 77.8%; bone marrow invasion predictive value, 92.9%; and cortical bone invasion predictive value, 58. 3%.

**Table 6:** Volumetric metabolic parameters as diagnostic and predictive parameters in mandibular invasion.

(A) MTV as a diagnostic and predictive parameter in mandibular invasion						
Parameter	Cutoff value	Prevalence (%)	Sensitivity (%)	Specificity (%)	PPV (%)	NPV (%)
MTV	2.9	85.1	97.5	71.4	95.1	83.3
(cm^3^)						
(B) TLG as a diagnostic and predictive parameter in vertical mandibular invasion (progression to the bone marrow or limitation to the cortical bone)						
Parameter	Cutoff value	Prevalence (bone marrow invasion rate) (%)	Sensitivity (%)	Specificity (%)	Bone marrow invasion predictive value (%)	Cortical bone invasion predictive value (%)
TLG	53.9	77.5	83.9	77.8	92.9	58.3
(bw/cm^3^)						

MTV = metabolic tumor volume, NPV = negative predictive value, PPV = positive; predictive value, TLG = total lesion glycolysis.

## 4. Discussion

Regarding the diagnosis of mandibular invasion by LGSCC, CT imaging was shown to be useful for the diagnosis of not only positive or negative invasion but also detailed vertical invasion, precisely, progression to the bone marrow or limitation to the cortical bone. However, it was found that diagnoses based on CT imaging alone could lead to underestimation and surgeries performed with inadequate vertical safety margins. Therefore, other diagnostic and predictive factors are needed to complement CT imaging for a more reliable diagnosis. The present study showed that MTV was an independent diagnostic and predictive factor for the presence or absence of mandibular invasion. In addition, TLG was an independent factor for vertical mandibular invasion, whether progression to the bone marrow or limitation to the cortical bone.

The usefulness of the SUV as a semi-quantitative indicator in ^18^F-FDG PET is known, and its maximum value, SUVmax, is usually used. However, because SUVmax is a value of only 1 voxel, it does not reflect glucose metabolism in the whole tumor and is characterized by a high value if the glucose metabolism in a limited area is high. Therefore, it is difficult to accurately determine tumor activity or residual activity after treatment based on SUVmax alone, as SUVmax is not indicative of overall tumor metabolism.^[29,30]^ In contrast, SUVpeak is a new parameter that measures the mean value of SUV within a 1 cm^3^ voxel in the VOI and shows the highest mean value, which is superior to SUVmax in terms of reproducibility and stability.^[31]^ However, SUVpeak also reflects only some of the tumor’s metabolic activity, and problems similar to that of SUVmax occur when tumor volume is large.^[32,33]^ Srinivas et al^[34]^ reported that accumulation on PET was dependent on tumor volume. In addition, recent advances in image analysis modalities and 3-dimensional display technology have enabled rapid and reliable assessment reflecting tumor volume. The usefulness of MTV and TLG, new volume-based parameters in ^18^F-FDG PET, have been shown. They represent alternative parameters to SUVmax by providing more relevant tumor information while combining both metabolic activity and 3-dimensional tumor volume. In other words, metabolic tumor volume parameters, such as MTV and TLG, are considered to be more suitable for assessing tumor activity than SUV values.

MTV and TLG have been reported to be useful for predicting recurrence, metastasis, and prognosis of head and neck cancer, including oral cancer, and in various other areas of cancer.^[5–8,35–38]^ Suzuki-Shibata et al,^[39]^ Lim et al,^[40]^ and Kim et al,^[41]^ reported the usefulness of metabolic volume-based parameters for predicting the efficacy of chemotherapy and molecular-targeted drug therapy. Recently, metabolic volume-based parameters have been considered for local diagnostic applications with regional specificity, such as the relationship between glucose metabolism and epithelial-mesenchymal interactions,^[42]^ the relationship between differentiation and clinical progression.^[43]^ In addition, their significance as diagnostic parameters for staging decisions have been revealed.^[44]^ Fukuhara et al^[45]^ and Bae et al^[46]^ pointed out that these parameters were useful for predicting the extranodal spread of cervical lymph node metastases.

The usefulness of metabolic parameters as diagnostic and predictive parameters for mandibular invasion in patients with oral cancer has been previously reported, but all studies evaluated using SUVmax alone.^[9,10]^ Additionally, no studies have applied metabolic parameters such as MTV or TLG. Koyasu et al^[47]^ assessed the relationship among MTV, TLG, and invasive potential in patients with head and neck squamous cell carcinoma. They showed that an increase in tumor metabolic capacity increases the migratory capacity of cancer cells, which in turn leads to an increase in invasive ability. Since MTV is mainly an indicator of volume factors, TLG, which reflects information on glucose metabolism, may be a better parameter for determining cancer invasiveness.^[48,49]^ Carcinomas with high glucose metabolism, specifically with high SUV, are highly invasive.^[9,50,51]^ In the present study, TLG was an independent factor for more detailed diagnosis and prediction in mandibular invasion, vertical mandibular invasion of LGSCC, supporting previous reports.

A decision analysis (2 × 2 contingency table) was performed for MTV which diagnoses the presence of mandibular invasion of LGSCC, and TLG which diagnoses vertical invasion, using cutoff values (MTV: 2.9 cm^3^, TLG: 53.9 bw/cm^3^) obtained from ROC. Furthermore, the usefulness as an imaging diagnosis was investigated. The results of MTV as a diagnostic and predictive parameter for mandibular invasion showed specificity of 97.5% and 83.9%, and PPV of 95.1% and 92.9%, respectively (Table 6). A recent study reported the results as, multi-slice CT: sensitivity: 85.71%, PPV: 89.47%, Denta-scan: 85.71% and 66.66%, and SPECT scan: 100% and 56%, respectively.^[3]^ These results were similar to the present study that showed a sensitivity of 84.3% and PPV of 91.5% (Table 3). The use of MTV as an adjunct to CT imaging was considered to provide a more reliable diagnosis. Furthermore, the sensitivity and PPV of TLG for the diagnosis of more detailed vertical bone invasion shown in this study were 83.9% and 92.9%, respectively, suggesting that the use of metabolic volume-based parameters as adjuncts to CT imaging may provide a more reliable diagnosis of mandibular invasion by LGSCC. A more accurate preoperative diagnosis of the extent of vertical mandibular invasion, whether progression to the bone marrow or limitation to the cortical bone, will lead to the selection of the appropriate surgical method for the mandibular bone.

There are 2 limitations of this study. First, PD-L1 did not show significant results to be a predictive factor in univariate analysis. There have been reports of PD-L1 and metabolic imaging parameters demonstrating correlation^[52–55]^ and no correlation.^[56,57]^ PET also provides information on the metabolic state of the tumor microenvironment (TME), as the glucose analogue ^18^F-FDG is transported by cancer cells and active immune cells, such as tumor-infiltrating lymphocytes (TIA) and tumor-associated macrophages (TAM). Therefore, hypermetabolism of ^18^F-FDG is not restricted to carcinomas, but is also observed in active inflammatory cells and immunocompetent cells. These were thought to be reflected in the accumulation on PET. The expression of PD-L1 in this study assessed cancer cells only, which could be the reason why MTV and TLG were extracted as independent factors for predicting mandibular invasion, but not PD-L1. In the TME, PD-L1 is also widely expressed on immune cells (such as CD4(+) T cells, CD8(+) T cells, Treg cells, B cells, Langerhans cells, and monocytes) and non-immune cells (such as vascular endothelial cells, and various mesenchymal cells) in addition to cancer cells. The precise relationship between MTV, TLG, and PD-L1 could be elucidated in future studies if the expression of PD-1 on these cells in the TME of the mandibular invasive areas is properly analyzed. Another problem is that the criteria for PD-L1-positive expression have not been consistent in previous reports, which is an issue for future research.

Second, in the present study, we specifically selected PTHrP from the perspective of the RANKL/RANK pathway,^[11–14,58]^ IL-6 from a perspective other than the RANKL/RANK pathway,^[59]^ and E-cadherin from a comprehensive perspective of the RANKL/RANK pathway and TME^[12,60,61]^ to investigate whether the expression of these proteins, using IHC, can be diagnostic and predictive factors for mandibular invasion (Table 1). However, neither protein expression was a significant diagnostic and predictive factor. The criteria for IHC used in the present study were in accordance with previous reports,^[19,26,27]^ but differences in staining of antibody-positive cells (cytoplasm or cell membrane) due to the experimental environment were considered problematic. Furthermore, the criteria for establishing diagnostic and predictive parameters, as well as PD-L1 expression, warrant future research. This study also revealed that the pathological diagnosis of mandibular invasion by IHC is currently not useful and still has to rely on morphological diagnosis, such as using INF.

## 5. Conclusion

This study demonstrated that in addition to morphological imaging by CT, assisted tumor volume-based metabolic parameters on ^18^F-FDG PET were important for predicting the presence of pathological mandibular invasion in patients with LGSCC. A more accurate preoperative diagnosis of vertical mandibular invasion, precisely, progression to the bone marrow or limitation to the cortical bone, would lead to selecting the appropriate surgical method for the mandible. Prevention of over-surgery, under-surgery, and additional treatment might contribute significantly to improving the QOL of patients. This is the first report showing the usefulness of volume-based metabolic parameters on ^18^F-FDG PET as diagnostic and predictive factors for more detailed vertical invasion. This study also revealed that the pathological diagnosis of mandibular invasion using IHC is currently not useful and still has to rely on morphological diagnosis such as using INF.

## Acknowledgments

The authors would like to thank clinical laboratory technician Koji Isoda for his help in preparing the specimens for this study. We would also like to thank Editage (www.editage.com) for English language editing.

## Author contributions

**Conceptualization:** Takahiro Shimizu, Mai Kim.

**Data curation:** Takahiro Shimizu, Citra R.A.P. Palangka.

**Formal analysis:** Takahiro Shimizu, Mai Kim.

**Investigation:** Mai Kim, Citra R.A.P. Palangka.

**Project administration:** Mai Kim, Satoshi Yokoo.

**Resources:** Mai Seki-Soda, Masaru Ogawa, Yu Takayama.

**Supervision:** Mai Kim, Satoshi Yokoo.

**Writing – original draft:** Takahiro Shimizu.

**Writing – review & editing:** Satoshi Yokoo.
